# Modified osteotomy of posterolateral overhanging part of the trochanter via posterior approach for hip arthroplasty: an anatomical study

**DOI:** 10.1186/s12891-020-3088-9

**Published:** 2020-02-24

**Authors:** Xiaoxiao Zhou, Houlin Ji, Jinhua Guo, Yang Yang, Pan Cai, Xianlong Zhang

**Affiliations:** 1 Department of Orthopedics, Shanghai University of Medicine & Health Sciences Affiliated Zhoupu Hospital, Shanghai, China; 2 Graduate School of Shanghai University of Traditional Chinese Medicine, Shanghai, China; 3 Department of Human Anatomy, Guangdong Medical University, Xincheng Road, Dongguan City, 523808 Guangdong Province China; 4 Department of Orthopedics, Taizhou Hospital of Zhejiang Province, Affiliated to Wenzhou Medical University, No. 150 Ximen Street, Linhai City, 317000 Zhejiang Province China; 5 Department of Orthopedics, Sixth People’s Hospital, Shanghai Jiao Tong University, Shanghai, China

**Keywords:** Posterolateral overhanging part of the trochanter, Osteotomy, Posterior approach, Arthroplasty

## Abstract

**Backgroud:**

The osteotomy of the posterolateral overhanging part (PLOP) of the greater trochanter via posterior approach has been used for the hip arthroplasty for decades with good results. However, the osteotomy method remains undefined and the precise adjacent structures around PLOP have not been reported. The purpose of this study was to present a modified PLOP osteotomy approach and perform a detailed study of the topographic and surgical anatomy of the PLOP.

**Methods:**

The peri-PLOP soft tissue and the bony parameters were measured using 10 cadavers with 20 hips and 20 skeletal hip specimens, respectively.

**Results:**

A 1.8-cm vertical osteotomy did not jeopardize the femoral neck, and a 1.8-cm wide bone block did not damage the insertions of the short external rotators. The average distances between the most distal branch of the superior gluteal nerve/artery and the 1.8-cm point of the greater trochanter were 5.70 ± 0.66 cm and 6.33 ± 0.56 cm, respectively.

**Conclusion:**

For osteotomy of the PLOP, we suggested that the width of the upper side from the lateral to medial greater trochanter should be 1.8 cm, depth of vertical osteotomy should be 1.8 cm, and length of the posterior edge should be 4 cm. Obturator externus tendon should be kept within the bone block of osteotomy. The proximal extension of the gluteus medius muscle split should be limited to 5.5 cm at the 1.8 cm-point of the greater trochanter.

**Level of evidence:**

Prospective comparative study Level II.

## Background

The posterior approach was widely used for hip arthroplasty because the excellent exposure of the acetabulum and posterior femur. However, the short external rotators require abscission, which may lead to prosthesis instability and a high rate of postoperative dislocations [1]. The injury of the posterior soft-tissue envelope of the hip, which prevents posterior dislocation and excessive internal hip rotation, is a major contributing factor. Repairing the posterior capsule and short external rotators can reduce the prosthesis dislocation incidence [2–4]. However, even if the obturator internus, obturator externus, and piriformis tendon are repaired, the dislocation rates of the posterior approach for arthroplasty are still higher than the rates of other approaches, resulting in a poor function [5–7].

Several techniques, such as muscle-sparing approaches and minimally invasive posterior approach with preservation of the external rotator muscle, were developed to descend the dislocation incidence of the posterior approach [8]. The long operative time consuming, limited visualization of surgical field, high incidence of component malposition, especially the steep and extended learning curves, impeding the popularity of posterior minimal invasion approach [9]. Hence, the classical posterior approach needs some modifications to avoid the injury of the short external rotators.

The proximal femoral attachment of gluteus medius, short external rotators, and a posterior capsule is located at the posterolateral overhanging part (PLOP) of the greater trochanter. It was reported that the PLOP osteotomy could provide good exposure and reserve the posterior soft-tissue of the hip, leading to a low rate of prosthesis instability and dislocation [10–12]. However, to our best knowledge, no studies have investigated the relationship between the PLOP and the short external rotators. The purpose of the current study was to present a modified PLOP osteotomy approach and perform a detailed study of the topographic and surgical anatomy of the PLOP.

## Methods

### Materials

Ten intact embalmed cadavers (20 hips) donated for medical education and research were collected, following approval by the institutional review board (IRB). The hips had no documented medical history and no findings of dissection that suggested pathology of the hip. The cadaver specimens were used to study the anatomy of the greater trochanter, short external rotators, and their neurovascular supply, especially the topography of the PLOP.

Another 10 adult bony specimens (20 hips) collected were used to measure the bony structures of the PLOP. The subjects did not have findings of dissection that suggested pathology of the hip. Details of the subjects’ ante-mortem ages, weights, and mobility statuses were unavailable. IRB approval was not required because no human subject information was gathered for this part of the study.

### Experimental procedures

**Fig. 1:**
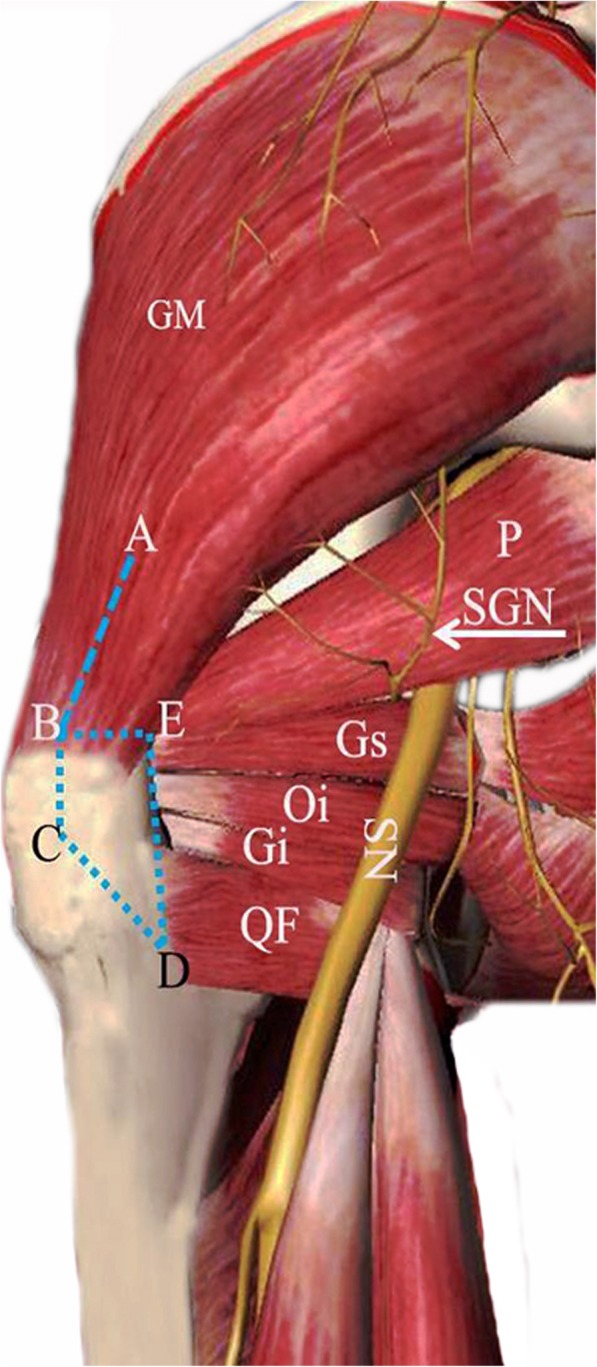
A schematic diagram of the posterior view of the left hip shows the short external rotator muscles and the mark of osteotomy of the PLOP of the greater trochanter in a three-dimensional body model. The bone block was approximately 1.8 cm from medially to laterally at its upper width (BE), and the posterior edge’s length was 4 cm (ED). The BC was approximately 2 cm and parallel to the line of the ED, and inclined posteroinferior to connect with point D. The depth of the bone block was 1.8 cm. Splitting the gluteus medius beyond the safe distance (AB) of 5.5 cm at the 1.8-cm point of the greater trochanter placed the most distal branch of the SGN at risk. GM: gluteus medius; P: piriformis; SGN: superior gluteal nerve; Gs: gemellus superior; Oi: obturator internus; Gi: gemellus inferior; QF: quadratus femoris; SN: sciatic nerve

To study the anatomy of the hip, we removed the skin, fat, and subcutaneous tissues from the lateral and posterior aspects of the hip and buttocks until only the gluteus medius, piriformis, obturator internus, obturator externus, gemellus superior, gemellus inferior, quadratus femoris, and capsular structures remained. During each dissection procedure, we identified the neurovascular structures as they related to the exposed structures (Fig. 1).

### Measurements

For the gluteus medius muscle, the maximum length and width, width at its attachment, location of the neurovascular pedicle as it exited the greater sciatic notch, and the distance of BE (Fig. 1) between the 1.8-cm point of the greater trochanter from medially to laterally and the neurovascular structures were measured. The origins and insertions, and their length, width, and thickness of the muscular and tendinous portions at their insertions of the short external rotators were recorded. The lengths of the tendons at their insertion in the short external rotator muscles covered by the PLOP were also measured. The PLOP formed a similar triangular structure, and the length of each side and distances from the cortex of the greater trochanter to the femoral neck at the points of osteotomy were measured.

A caliper was used to measure the length of the muscles, tendons, and bone. Measurements are reported in centimeters (cm) as the mean ± standard deviation and range.

### Statistical analyses

Paired *t*-tests were used to compare numeric variables, and the Bonferroni multiple comparisons test of one-way analysis of variance was used to compare multiple groups; if equal variances were not assumed, the Dunnett *t-*3 test was used to compare differences between the groups. Data are presented as mean ± standard deviation. A *p*-value <0.05 was considered statistically significant. Statistical analyses were performed using SPSS, version 23 (IBM Corp., Armonk, NY, USA).

## Results

The mean age of 10 cadaveric specimens at the time of death was 75.4 ± 7.08 years (range: 63–87 years). Details of their ante-mortem weight and mobility status were unavailable, but their mean estimated height was 162.9 ± 9.24 cm. The main demographic data obtained from the 10 cadaveric specimens were summarized in Table 1. There was no significant difference between male and female cadaveric specimens in age (*p* = 0.6), but the male cadaveric specimens had a significantly higher estimated height than the female ones (*p* < 0.001).

**Table 1:** Main demographic data obtained from 10 cadaveric specimens (20 hips)

	Male cadaveric specimens (*n* = 5)	Female cadaveric specimens (*n* = 5)	*P*-value
Age (years)	74.40 ± 9.61(63–87)	76.40 ± 3.37 (72–81)	0.6
Estimated height (cm)	171.00 ± 3.59 (165–175)	154.80 ± 4.64 (150–161)	<0.001

All values are expressed as the mean ± standard deviation. Differences are considered significant at *p* < 0.05

The m. gluteus medius originated from the lateral surface of the ilium, and it attached to the whole superior margin of the greater trochanter. The width at the insertion point on the greater trochanter was approximately 4.81 ± 0.68 cm (range: 3.88–5.84 cm) (Table 2).

**Table 2:** Measurements of the GM muscle and distances between the ITC and SN

Measurement	Mean ± standard deviation (cm)	Range (cm)
Length	12.12 ± 1.26	10.26–14.02
Width	12.38 ± 0.65	11.48–13.36
Width of the insertion	4.81 ± 0.86	3.88–5.84
T-GM, split–SGN (1.8 cm)	5.70 ± 0.66	4.54–6.62
T-GM, split–SGA (1.8 cm)	6.33 ± 0.56	5.66–7.92
Upper distance	2.22 ± 0.20	1.89–2.52
Middle distance	1.90 ± 0.31	1.30–2.35
Lower distance	1.97 ± 0.14	1.27–2.16

*GM* Gluteus maximus, *ITC* Intertrochanteric crest, *SN* Sciatic nerve, *T-GM* Transgluteal maximus; upper, middle, and lower mean distance from the intertrochanteric crest to sciatic nerve, *SGN* Superior gluteal nerve, *SGA* Superior gluteal artery

The sciatic nerve descended through the greater sciatic notch behind the intertrochanteric crest. The distance between the intertrochanteric crest and sciatic nerve was 2.22 ± 0.20 cm in the apex, 1.90 ± 0.31 cm in the middle, and 1.97 ± 0.14 cm at the bottom (Table 2).

The superior gluteal nerve (SGN) passed from the greater sciatic notch through the space between the m. obturator internus and m. gluteus minimus. The SGN generated one to three branches along its path, and the average distance between the most distal branch of the SGN and the 1.8-cm point of the greater trochanter was 5.70 ± 0.66 cm (range: 4.54–6.62 cm) from medially to laterally. The average distance from the 1.8-cm point to the superior gluteal artery (SGA) was 6.33 ± 0.56 cm (range: 5.66–7.92 cm) (Table 2). The SGN and SGA had branches that supplied the m. gluteus medius and m. gluteus minimus at numerous locations along their lengths.

The piriformis muscle originated from the anterior surface of the ipsilateral half of the second to fourth sacral vertebrae. It descended laterally to leave the pelvis through the greater sciatic notch, and it attached to the upper border of the greater trochanter with a column shape, anterosuperior to the trochanteric fossa. The distance from the lateral margin of the sacrum to the tendon’s insertion into the greater trochanter was defined as the length, and the length, the width and the thickness of the piriformis tendon were 2.28 ± 0.13, 0.30 ± 0.06 and 0.30 ± 0.06 cm, respectively. No tendon at its insertion was covered by the PLOP of the greater trochanter (Table 3).

**Table 3:** Measurement of the short external rotators

Muscle	Muscle length (cm)	Muscle width (cm)	Tendon length (cm)	Tendon width (cm)	Tendon thickness (cm)	Covered part (cm)
Piriformis	7.29 ± 0.19	2.69 ± 1.12	2.28 ± 0.13	0.30 ± 0.06^a^	0.30 ± 0.06^b^	
Obturator externus	3.75 ± 0.59	2.14 ± 0.21	4.67 ± 0.35	0.74 ± 0.18	0.41 ± 0.11	1.13 ± 0.27
Gemellus superior	3.91 ± 0.47	0.72 ± 0.16	2.46 ± 0.28	0.81 ± 0.25	0.43 ± 0.09	1.16 ± 0.29
Obturator internus	10.12 ± 0.63	2.16 ± 0.21	4.13 ± 0.33	0.81 ± 0.25	0.43 ± 0.09	1.16 ± 0.29
Gemellus inferior	4.22 ± 0.35	0.69 ± 0.18	0.75 ± 0.11	0.81 ± 0.25	0.43 ± 0.09	1.16 ± 0.29
Quadratus femoris	5.16 ± 0.18	3.12 ± 0.34				

The conjoined tendon includes the gemellus superior, obturator internus, and gemellus inferior. ^a^The width of the tendon of the piriformis versus [vs.] that of the obturator externus or conjoined tendon, *p* < 0.001; obturator externus vs. conjoined tendon, *p* = 0.724. ^b^The thickness of the tendon of the piriformis vs. that of the conjoined tendon, *p* < 0.001; obturator externus vs. conjoined tendon, *p* = 0.325. All values are expressed as the mean ± standard deviation. Differences are considered significant at *p* < 0.05

The obturator internus tendon originated from the obturator canal via the lesser sciatic notch and was connected to the superior and inferior gemelli, both of which originated from the lesser sciatic notch. Three of these tendons formed a conjoined tendon, and flattened and entered the superior margin and entire medial surface of the greater trochanter close to the proximal part of the anterior intertrochanteric line. The three tendons could not be completely separated after they were joined. The width and thickness of the conjoined tendon were 0.81 ± 0.25 and 0.43 ± 0.09 cm, respectively, and the covered part of the conjoined tendon at its insertion was approximately 1.16 ± 0.29 cm (range: 0.82–1.55 cm) (Table 3).

The obturator externus muscle originated from the anterolateral surface of the pubic ramus. Parts of the tendon were located behind the quadratus femoris, and the flat-shaped end was attached independently, posteroinferior to the conjoined tendon in the pyriform sinus. The length, width, and thickness of the obturator externus tendon were 4.67 ± 0.35, 0.74 ± 0.18, and 0.41 ± 0.11 cm, respectively. The covered part of the tendon was approximately 1.13 ± 0.27 cm (range: 0.82–1.55 cm).

The width of the obturator externus tendon and conjoined tendon were significantly larger than those of the piriformis tendon (*p* < 0.001), but there were no significant differences between the obturator externus and conjoined tendon (*p* = 0.724). The thickness of the tendon of the piriformis was significantly thinner than that of the obturator externus or conjoined tendon (*p* < 0.001), but there were no significant differences between the obturator externus and conjoined tendon (*p* = 0.325) (Table 3).

The quadratus femoris originated from the ischial tuberosity and attached at one-third of the intertrochanteric crest and part of the lesser trochanter. The length and width of the muscle for all the short external rotators are displayed in Table 3.

Regarding the length and width of the m. gluteus medius and external rotator muscles, there were significant differences (*p* < 0.05) between the sexes (data not shown), which we ascribed to the significant difference of height between the male and female cadaveric specimens.

**Fig. 2:**
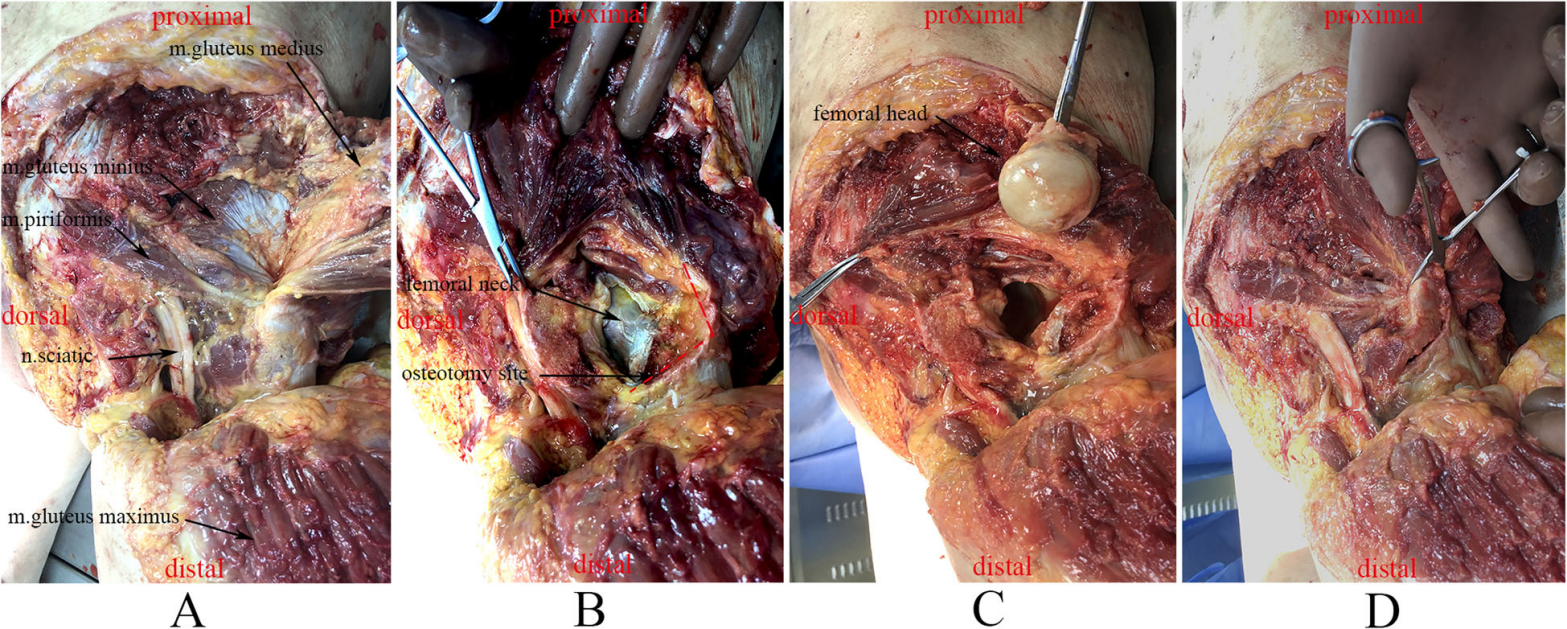
(**a**) shows the abutment relationship of soft tissues around the posterolateral overhanging part (PLOP) of the greater trochanter; (**b**) shows the osteotomy of PLOP (dash line); (**c**) shows the femoral head osteotomy could be performed easily with the osteotomy part of PLOP reflected up; (**d**) shows that after the replacement of hip, osteotomy part of PLOP could be restored anatomically

**Fig. 3:**
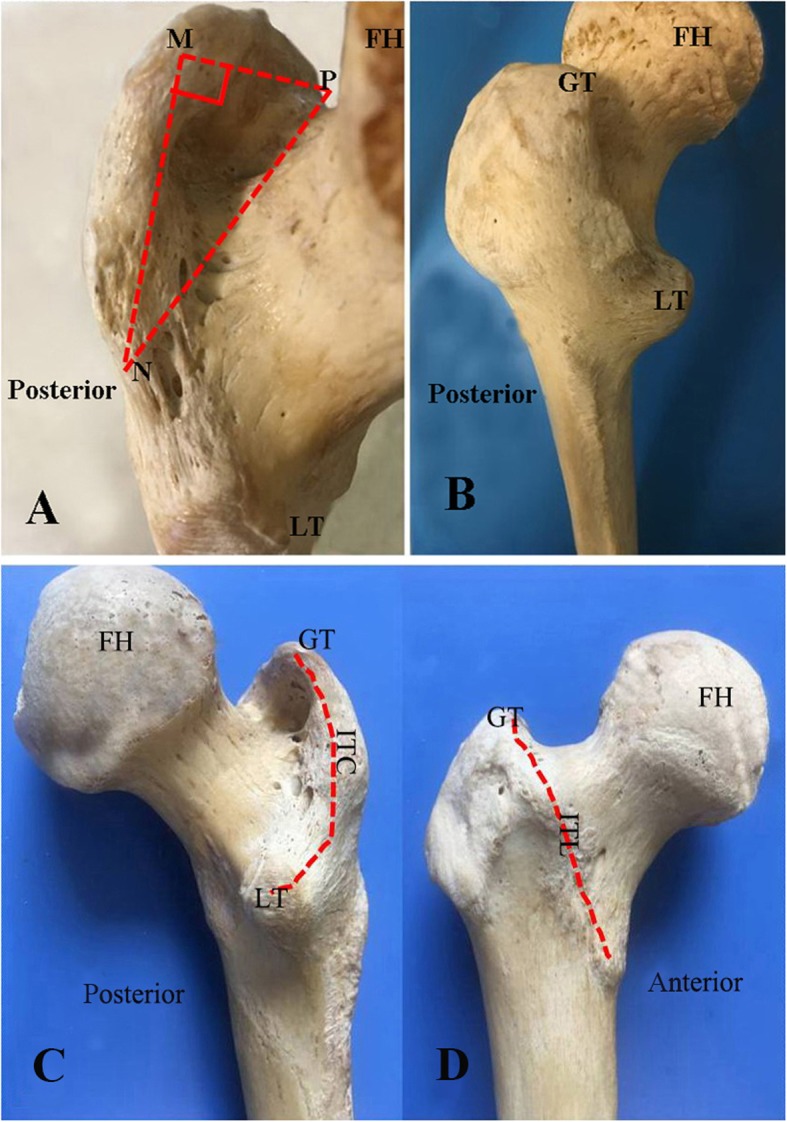
The posterosuperior view of a cadaveric specimen of the hip shows the posterolateral overhanging part (PLOP) of the greater trochanter (GT). **a** shows the right triangular structure formed by the PLOP, M angle (tip of the GT) is its right angle, NP is the base of the triangle, and the posterior (MN) and upper (MP) border are the other two sides. The O point is the pyriform sinus. **b** and **c** show the posterior view of the proximal femur. **c**: The anterior view of the proximal femur is displayed. The red dash line represents the intertrochanteric crest (ITC). **d** shows the anterior view of a cadaveric specimen of the hip. The red dash line represents the ITL. LT: lesser trochanter; ITL: intertrochanteric line; FH: femoral head

The PLOP was located at the posterosuperior part of the greater trochanter, with the pyriform sinus as its center, and part of the intertrochanteric crest extended upward and formed the posterior upper structure as the attachment of the gluteus medius and most of the short external rotators’ tendons. The femoral neck was located under the inferior PLOP (Fig. 2 and Fig. 3). PLOP was a right triangular like structure with the tip of the greater trochanter as its right angle (Fig. 3). The lengths of the superior border, posterior border, and triangular base and depth of the bone block are showed in Table 4.

**Table 4:** Measurements of the PLOP of the greater trochanter

PLOP anatomical parameter	Mean ± standard deviation (cm)	Range (cm)
Length of the posterior edge	4.00 ± 0.27	3.67–4.38
Length of the upper edge	3.37 ± 0.13	3.14–3.56
Length of the triangle’s base	4.92 ± 0.67	2.17–5.29
Depth at the 1.8-cm point	1.81 ± 0.04	1.74–1.89

*PLOP* Posterolateral overhanging part

## Discussion

The PLOP has been mentioned in previous studies with excellent long-term results and patient satisfaction [10–12], and it consists of posterosuperior extension along with the intertrochanteric crest, forming a bony structure that overhangs the greater trochanter. The PLOP and its attached soft tissues play a major role in preventing hip dislocation. Iyer first devised the posterior approach combined the osteotomy of the PLOP in 1981 [12]. The technique was modified by Sanchez-Sotelo et al. and has been used for decades by his team. Although good clinical outcomes have been achieved with this technique, including a high union rate and low frequency of late instability [10]. But there was no detailed anatomical studies of the PLOP until now.

In our study, we found that the PLOP had a right triangular structure, and the piriformis tendon around the PLOP is independent and not joined to the obturator internus; moreover, this tendon attached anterosuperiorly to the greater trochanter, not the anterior intertrochanteric line, as mentioned in a previous study [13]. Few studies about the anatomy of the m. obturator externus neglected the importance of hip stability [7]. Since the tendon of the obturator externus was located at one of the weakest areas of the hip joint capsule, it was speculated that these weak areas of the hip joint capsule could be reinforced by the tendon of the obturator externus [14, 15]. Because this muscle has a larger mass and key location among the short external rotator muscles, we inferred that it may play a more important role in maintaining hip stability, and it should be kept within the osteotomy of the bone block.

According to a previous study, the superior side of the osteotomy of the bone block was 1 cm wide from medially to laterally [10] or the posterior one-third of the gluteus medius [11, 12]. Overall consideration of the tendon insertions of the conjoined tendon and nearby bony structures is necessary to ensure bone-to-bone contact after fixing the prosthesis. On the basis of our data, we recommend that the superior side of the bone block of the greater trochanter should be 1.8 cm wide, which could protect the tendon insertions of the short external rotators as a whole.

Another important point was the length for the osteotomy of the bone block. A smaller bone block means insufficient bone contact, which may increase the nonunion rate. Whereas, a large bone block may jeopardize the inner aspect of the femoral neck, resulting in an unstable femoral prosthesis postoperatively. No precise data about the length of the posterior edge has been provided in any studies so far; a small bone block may only include the tip of greater trochanter [11, 12] or part of the insertion site of the gluteus medius, piriformis, and conjoined tendon [10]. Based on the important role of the tendon of obturator externus and the facts that the PLOP should include the insertion of the tendon of obturator externus and bone block should as large as possible without damaging the inner aspect of the femoral neck, a 4-cm long osteotomy of the bone block was recommended. Our results are similar to Sanchez-Sotelo et al. [10], although their findings were not as clearly described. According to Sanchez-Sotelo et al.’s report [10], the inner aspect of vertical osteotomy should exist just posterior to the femoral neck. The transverse portion of the osteotomy should be angled slightly from inferolaterally to superomedially. There were no accurate data about the depth of osteotomy. The data of our study suggest that a 1.8-cm vertical osteotomy does not damage the femoral neck just under the joint capsule and above the cortex of it, so we recommend a 1.80-cm deep bone block (Fig. 1 and Fig. 2).

Another key point was the distance between the SGA/SGN and the greater trochanter. The relative location of the most distal branch of the SGN with respect to the gluteus medius muscle has been emphasized in numerous publications and anatomic studies. Most investigators have reported that the safe distance relative to the greater trochanter was 5 cm [16, 17]. However, the maximum distance of the split from the greater trochanter depends on several factors, including the size of the patient, sex (and sometimes race), and anatomic location of the split itself relative to the tip of the greater trochanter. Herein, using the 1.8-cm point of the greater trochanter from laterally to medially as a reference point, the branches of the SGA and SGN were located close to 5.70 cm cephalad. Splitting the m. gluteus medius beyond the safe distance of 5.5 cm at the 1.8-cm point of the greater trochanter places the most distal branch of the SGN at risk. Clinically significant muscle dysfunction caused by damage to the SGN is observed less frequently [18].

Because of the extensile nature of and the surgeon’s familiarity with the standard posterior approach to the hip, efforts have been made to use this approach to preserve the external rotator musculature. The most biologically effective approach that can maximize surgical exposure of the entire acetabulum and femur is the external rotator sparing approach and knowing the posterior approach well. We believe that osteotomy of the PLOP provides a versatile alternative to the currently popular minimally invasive approaches without pigeonholing the surgeon into using other less extensive procedures.

This study had several limitations. Firstly, a relatively small sample size; Secondly, instead of fresh specimens, embalmed cadavers were used which may result in an inaccurate adjacent relationship of soft-tissue around PLOP. Thirdly, we did not obtain detailed information regarding the body size of subjects before their death. Fourthly, the study population was limited to one area of a single country. Ethnic differences in bony geometry may result in variations in the characteristics of tendon attachments. Finally, we did not perform cutting study to show the osteotomy of PLOP can prevent dislocation in a cadaveric study. So, the results of the present study must be interpreted with some caution, and additional studies involving large quantity specimen and precise clinical studies are required to confirm the effectiveness of this method.

## Conclusion

For osteotomy of PLOP, we suggest that the width of the upper side from the lateral to medial greater trochanter should be 1.8 cm, depth of vertical osteotomy should be 1.8 cm, and length of the posterior edge should be 4 cm. Obturator externus tendon attachment should be reserved within the bone block of osteotomy. The proximal extension of the gluteus medius muscle split should be limited to 5.5 cm at the 1.8 cm-point of the greater trochanter. Further research is required to determine the veritable advantages of performing osteotomy of the PLOP for the posterior approach.

## Data Availability

The datasets used and/or analysed during the current study are available from the corresponding author on reasonable request.

## Abbreviations

PLOP: Posterolateral overhanging part
SGA: Superior gluteal artery
SGN: Superior gluteal nerve
